# Improved Mineral Acquisition, Sugars Metabolism and Redox Status after Mycorrhizal Inoculation Are the Basis for Tolerance to Vanadium Stress in C3 and C4 Grasses

**DOI:** 10.3390/jof7110915

**Published:** 2021-10-27

**Authors:** Samy Selim, Walid Abuelsoud, Salam S. Alsharari, Bassam F Alowaiesh, Mohammad M. Al-Sanea, Soad Al Jaouni, Mahmoud M. Y. Madany, Hamada AbdElgawad

**Affiliations:** 1 Department of Clinical Laboratory Sciences, College of Applied Medical Sciences, Jouf University, Sakaka 72341, Saudi Arabia; 2 Department of Botany and Microbiology, Faculty of Science, Cairo University, Giza 12613, Egypt; WalidAbuelsoud@sci.ca.edu.eg (W.A.); madany@sci.cu.edu.eg (M.M.Y.M.); 3 Biology Department, College of Science, Jouf University, Sakaka P.O. Box 72341, Saudi Arabia; sssharary@ju.edu.sa (S.S.A.); bfalawish@ju.edu.sa (B.F.A.); 4 Pharmaceutical Chemistry Department, College of Pharmacy, Jouf University, Sakaka 72341, Aljouf Province, Saudi Arabia; mmalsanea@ju.edu.sa; 5 Hematology/Pediatric Oncology, Yousef Abdulatif Jameel Scientific Chair of Prophetic Medicine Application, Faculty of Medicine, King Abdulaziz University, Jeddah 21589, Saudi Arabia; saljaouni@kau.edu.sa; 6 Biology Department, College of Science, Taibah University, Al-Madinah Al-Munawwarah 41411, Saudi Arabia; 7 Botany and Microbiology Department, Faculty of Science, Beni-Suef University, Beni-Suef 62511, Egypt; 8 Integrated Molecular Plant Physiology Research, Department of Biology, University of Antwerp, 2000 Antwerp, Belgium

**Keywords:** antioxidant biosynthesis, mycorrhiza, primary metabolism, vanadium stress, redox status, tolerance

## Abstract

Vanadium (V) can be beneficial or toxic to plant growth and the interaction between arbuscular mycorrhizal fungi (AMF) and V stress was rarely investigated at physiological and biochemical levels of plant groups (C3 and C4) and organs (roots and shoots). We tested the potential of AMF to alleviate the negative effects of V (350 mg V/Kg soil) on shoots and roots of rye and sorghum. Relative to sorghum (C4), rye (C3) showed higher levels of V and lower levels of key elements under V stress conditions. V inhibited growth, photosynthesis, and induced photorespiration (increased HDR & GO activities) and oxidative damage in both plants. AMF colonization reduced V stress by differently mitigating the oxidative stress in rye and sorghum. This mitigation was accompanied with increases in acid and alkaline phosphatase activities in plant roots and increased organic acids and polyphenols exudation into the soil, thus reduced V accumulation (29% and 58% in rye and sorghum shoot, respectively) and improved absorption of mineral nutrients including Ca, Mg and P. AMF colonization improved photosynthesis and increased the sugar accumulation and metabolism. Sugars also acted as a supplier of C skeletons for producing of antioxidants metabolite such as ascorbate. At the antioxidant level, rye was more responsive to the mitigating impact of AMF. Higher antioxidants and detoxification defence system (MTC, GST, phenolics, tocopherols and activities of CAT, SOD and POX) was recorded for rye, while sorghum (C4) improved its GR activity. The C3/C4-specificity was supported by principal component analysis. Together, this study provided both fundamental and applied insights into practical strategies to mitigate the phytotoxicity hazards of V in C3 and C4 grasses. Moreover, our results emphasize the importance of AMF as an environment-friendly factor to alleviate stress effects on plants and to improve growth and yield of unstressed plants.

## 1. Introduction

Vanadium ranks 22nd among elements in the crust of the earth and it is the fifth most abundant transition element that is naturally found in limestones associated with other elements like iron oxide and in organic debris [1]. The pentaoxide form of vanadium is the most commonly existing, stable and the most mobile form. Vanadium is emitted into the environment via natural (like volcanoes, forest fires, atmospheric dust and biogenic processes) as well as through industrial practices (like steel industries, mining, metallurgy, galvanization, phosphate treating factories, petrochemical industries, burning of fossil fuels and application of fertilizers) [2,3]. Vanadium is one of the non-essential elements for plants that can be beneficial for growth and yield, however, when its concentration within plant tissues exceeds a certain threshold concentration, it is detrimental to plant metabolism and eventually retards growth and productivity [1]. Absorption of vanadium by plant roots does not require metabolic energy but it is pH-dependent i.e., its absorption is highest at pH value 4 and very low at soil pH 10 [1]. Moreover, vanadium can be taken up and metabolized in nitrogen-poor soils via nitrogen-fixing microorganisms like *Azotobacter vinelandii*, therefore vanadium is becoming available to the microbe-associated plants [1,4]. When available to plants, vanadium is highly retained within root tissues because, once it is absorbed, it becomes reduced and highly immobile [1]. For instance, in rape (*Brassica juncea*), it was observed that vanadium accumulation in tissues was highest in the roots, and the least in the stem, leaves and seeds [5]. Vanadates (vanadium oxides) can compete with phosphate for absorption by roots. Thus, the negative impact of vanadium on a plant’s biology is partly due to the structural analogy between vanadate and phosphate [6].

In nitrogen poor soils, trace amounts of vanadium stimulate the growth of nitrogenase activity in nitrogen fixing bacteria [4,7]. A 5 mM of ammonium vanadate in a hypotonic culture improved the growth, flowering, content of amino acids and sugars in roots and leaves of pepper [8]. Similarly, 250 ng/mL ammonium vanadate stimulated growth and chlorophyll content in tomato [9]. Above a certain threshold concentration, vanadium imposed a detrimental effect on the physiological and biochemical processes. At 15 mg/L and higher, vanadium in a hydroponic culture negatively affected the growth, photosynthesis and imposed oxidative stress in rice seedlings [10]. Toxic levels of vanadium in a growing medium imposes leaf chlorosis and necrosis and alters the electric potential of the cell membrane and disturbs absorption of the essential elements by the roots [11,12]. Vanadium also induces reactive oxygen species (ROS) production and accumulation by activating expression of the *respiratory burst oxidase* genes and by scavenging the cellular nonenzymatic antioxidants [1,13]. Vanadium stress has also been correlated with chromosomal aberrations, DNA fragmentation and inhibition of cell division [1,14,15]. Overall, high vanadium bioaccumulation in plants can exert toxic consequences such as inhibition of plant growth and photosynthesis, induction of oxidative damages and disruption of mineral homeostasis. Thus, it is very important to understand the mechanism of vanadium uptake and the extent of phytotoxicity in different plant species, which may help in regulating plant tolerance against vanadium stress.

Arbuscular mycorrhizal fungi (AMF) associate with many plant species and facilitate plant growth and enable them to cope the adverse environmental conditions [16]. AMF form hyphae, vesicles and arbuscules within plant roots. This network of root-hyphae enhances roots access to a larger volume of soil and hence can absorb larger amounts of water and nutrients and eventually improve plant growth and yield [17,18]. Moreover, soil enrichment with mycorrhiza improves soil physical characteristics and reduces soil erosion [19]. In some cases, mycorrhizal association with the host plant is essential for the survival of the plant [20]. AMF have been used to improve plant tolerance to soil contamination with heavy metals. For example, wheat association with AMF restored its growth under aluminum toxicity [21]. Moreover, AMF can bioremediate soils contaminated with heavy metals such as Cd, thus reducing their absorption by the plant roots [22]. Accordingly, AMF are very promising for sustainable agriculture for improving productivity and coping with the abiotic stresses.

AMF can facilitate phosphorus uptake and transport [23] that can reduce the competition between vanadium and phosphate. Although AMF and plant interactions have been explored under the stress of a wide range of toxic and heavy metals, the effects of vanadium on AMF-plant interaction as well as the potential role of AMF in inducing plant tolerance against vanadium stress have not been well-studied [1].

Giving the fact that increasing concentration of vanadium in soil altered plant growth, physiology, metabolism and mineral nutrition, the detailed mechanism of vanadium-induced toxicity should be adequately explored. Thus, the aim of this study was to precisely understand how vanadium accumulation in different plant species groups and plant organs affects root and shoot growth, physiology and biochemistry of rye (*Secale cereal* L., C3) and sorghum (*Sorghum bicolor* L., C4) plants. Furthermore, it was to explore the potentiality of a well characterized beneficial arbuscular mycorrhizal fungus (*Rhizophagus irregularis*) to alleviate the potential negative effects of vanadium on plants. Our hypothesis is that the chosen level of vanadium treatment could impose negative impact on the growth and physiology of both plant species, but through affecting different physiological and biochemical aspects that are different in the C3 and C4 plants. A study like this one is of paramount importance, especially with the increasing industrialization and urbanization rates in developing countries like Egypt with all of its consequent pollution potentials.

## 2. Materials and Methods

### 2.1. Plant Materials and Growth Conditions

Vanadium (V) as Na_3_VO_3_ was spiked in the soil potting mixture (clay:sand in 1:3 ratio) to measure its effect on plant growth. A 350 mg of Na_3_VO_3_ per kg of soil was selected to excrete stress based on preliminary experiments as it induces a reduction in a growth by about 50%. The soils have been screened with waterproof bags and aged for three months in pots to achieve a spike V partition balance (V). Spiked and not spiked soils then received a concentration of 150 spores per soil in a pot with pure trade inoculum of *Rhizophagus irregularis* (MUCL 41,833 obtained by Glomeromycota In vitro harvest (GINCO). Non-inoculated and not spiked soil served as the control treatments. Seeds of rye (*Secale cereale*) and sorghum (*Sorghum bicolor*) were sown and pots were kept in a monitored greenhouse at 21/18 °C, 16/8 h a day/night and 60% humidity and were regularly watered. After six weeks, the plants were harvested, and the roots and shoots were separated, thoroughly washed with deionized water and gently surface dried. Samples were immediately frozen in liquid nitrogen and stored at −70 °C for various biochemical analyses. Other shoots and roots were dried at 75 °C for 72 h to determine the dry matter and mineral elements. The percentage (%) of changes in FW and DW was calculated as ((Treatment − Control)/Control) × 100.

### 2.2. Colonization and AMF Growth Analysis

Successful colonization of mycorrhiza was verified following Phillips and Hayman [24]. The fresh roots (ca. 0.5 g) were clarified by heating at 90 °C in 10% potassium hydroxide (*w*/*v*) for 2 h, then washed with fresh potassium hydroxide and immersed in a mix of potassium hydroxide (10%) and hydrogen peroxide (10% *v*/*v*) in a ratio of 1:1 (*v*/*v*), then stained with 0.05% trypan blue in lactoglycerol. A stereomicroscope (40×) was used to display the stained roots, while the colonization rate was calculated by using the gridline intersect method [25]. Hyphal length was determined in rhizosphere and hyphosphere soils by the method of Andrade, Mihara [26].

### 2.3. Determination of Minerals Content

Oven-dried plant tissues (0.1 g) were digested in 5:1 *v/v* (HNO_3_/H_2_O) solution in an oven until clear solution was obtained. Element concentrations in the clear digestate were determined by mass spectroscopy (ICP-MS, Bremen, Germany) according to Hamad, AbdElgawad [27].

### 2.4. Photosynthetic Rate

The photosynthetic rates in mature leaves saturated with light was measured using LI-COR LI- 6400 (LI-COR Inc., Lincoln, NE, USA) as described [28]. Photochemical efficiency (Fv/Fm) of PSII was measured after 30 min in dark-adapted leaves with a fluorimeter (PAM2000, Walz, Effeltrich, Germany). Chlorophyll and carotenoids were extracted in 80% acetone and measured according to [29].

### 2.5. Root Phosphatase Activities, Citrate and Proline Content

Root tissue of rye and sorghum samples were weighted out and grinded in liquid nitrogen. Samples were extracted in universal buffer (MUB) [100 mM Tris, 100 mM maleic acid, 5 mM citric acid, and 100 mM boric acid] at pH 5.5 (acid phosphatase) and pH 8.5 (alkaline phosphatase). The reaction mixture consisted of MUB, and 3 mM *p*-nitrophenyl phosphate. The mixture was incubated at 37 °C. The reaction stopped by the addition of CaCl_2_ and NaOH. After centrifugation at 12,000× *g* for 15 min, the *p*-nitro- phenol content of the supernatant was quantified spectrophotometrically at 400 nm and compared to a standard concentration series of authentic p-nitrophenol.

Citric acid was extracted in 0.1% phosphoric acid-containing butylated hydroxyanisole and measured by HPLC (LaChom L-7455 diode array, LaChrom, Tokyo, Japan). Proline was extracted by homogenizing 100 mg finely ground liquid nitrogen-frozen fresh tissues in 1 mL of 3% aqueous sulfosalicyclic acid, followed by centrifugation at 10,000× *g* at 4 °C for 10 min. The clear supernatant was used to estimate proline content by acid ninhydrin method according to Bates, Waldren [30].

### 2.6. Extraction and Assessment of Sugars and Their Key Metabolizing Enzymes

Soluble sugars were extracted according to AbdElgawad, Peshev [31] by homogenizing finely ground 0.2 g fresh tissues in 1 mL of 50 mM TAE buffer pH 7.5 (0.02% sodium azide, 10 mM mannitol, 0.1% polyclar, 10 mM NaHSO3, 1 mM mercapto-ethanol, 1 mM phenylmethanesulfonylfluoride (PMSF)). The extract was clarified by centrifugation at 14,000× *g* for 5 min at 4 °C). A 150 μL of clear supernatant was heated for 5 min in a water bath at 90 °C. After cooling and centrifugation (14,000× *g*, 4 °C, 5 min), the supernatant was added to a mixed bed Dowex column (300 μL Dowex H+, 300 μL Dowex Ac–; both 100–200 mesh; Acros Organics, Morris Plains, NJ, USA). The column was eluted with 150 μL of ddH2O for six times. Glucose, fructose, sucrose concentrations were measured by HPAEC-PAD as before [32]. Total soluble sugars concentration in the total soluble extract was estimated by the anthrone reagent method according to Hodge and Hofreiter [33].

About 0.2 g of frozen shoots were used to extract sugars metabolizing enzymes in 1 mL HEPES buffer (100 mM HEPES pH 8.2, 10 mM EDTA, 15 mM KCl, 5 mM MgCl_2_, 2 mM sodium diethyl dithiocarbamate, 5 mM β-mercaptoethanol, 1% PPV) [34]. After centrifugation (14,000× *g*,4 °C, 15 min), the supernatant was conveyed to a clean tube for further analyses. Invertase activity in the supernatant was determined in TAE buffer (pH 8.5 with 100 mM sucrose). Reaction mixtures were kept at 30 °C, then the reactions were terminated by incubating in a boiling water bath for 5 min. The cell wall invertase was assayed from the pellet left after extracting soluble invertase by washing the pellet with ice-cold 50 mM Na-acetate buffer, pH 5.0 three times and then resuspending the pellet in the same buffer. An aliquot of the suspension was used to assay cell wall invertase as mentioned above. Sucrose phosphate synthase activity was measured by following sucrose synthesis in 1 mL of HEPES buffer (20 mM HEPES, pH 8.2, 2.2 mM UDP- glucose, 4.4 mM fructose-6-phosphate, 1 mM MgCl_2_, 2 mM NaF) at 37 °C for 15 min and stopped by adding 30% NaOH and 10 min boiling. The resulting sucrose was measured by the anthrone method mentioned above [31].

### 2.7. Quantification of Detoxification-Related Parameters

Glutathione-S-transferase (GST) was extracted in 50 mM potassium phosphate buffer (pH 7.0) and assayed following Mozer, Tiemeier [35]. Extraction and determination of metallothioneins (MTC) was done according to [36]. Briefly, samples were homogenized in 200 mM phosphate buffer (pH 7.2) for 30 min at 90 °C then clarified with centrifugation (16,000× *g* for 30 min at 4 °C) and MTC content was estimated electrochemically using the differential pulse voltammetry Brdicka reaction in the clarified supernatant. The total non-protein thiols were extracted in 5% sulfosalicylic acid, mixed with Ellman’s reagent and quantified spectrophotometrically at 412 nm [37]. The total phytochelatins (PC) content was estimated from the difference between the total non-protein thiol and total glutathione (GSH) content.

### 2.8. Quantification of Total Vanadium

A known weight of dried plant sample was digested in a known volume of 8:1:1 mixture of HNO_3_, H_2_SO_4_ and HClO_4_ at 120–130 °C for 5 h [38]. After cooling, the clear aliquot was diluted to 25 mL with deionized water for V analysis. To quantify total soil V, a 0.1 g soil fraction was heated with a mixture of concentrated HF, HNO_3_ and HClO_4_ [in ratio of 12:1:2 (*v*/*v*)] until completely dissolved. All measurements were made by a Perkin-Elmer spectrophotometer atomic absorption model Analyst 800 (USA). A standard stock solution containing 1 g L^−1^ V was used for preparing standard solutions of 10, 20, 40, 80, 160 and 200 μg V L^−1^ in the corresponding sample matrix.

### 2.9. Lipid Peroxidation Determination

Lipid peroxidation was quantified as MDA in 200 mg frozen shoot tissues, homogenized in 2 mL 80% ethanol by mortar and pestle. After centrifugation at 10,000× *g* for 15 min at 4 °C, the supernatant was incubated with thiobarbituric acid (TBA), to produce thiobarbituric acid-malondialdehyde (TBA-MDA). Absorbance was measured at 440, 532 and 600 nm in a 96-well plate reader [39].

### 2.10. H_2_O_2_ Concentration Quantification

H_2_O_2_ concentration was measured by the xylenol orange-based FOX1 method [40]. Specificity for H_2_O_2_ was tested by eliminating H_2_O_2_ from the reaction mixture with catalase. 

### 2.11. Total Antioxidant Capacity

Frozen tissues (200 mg FW) were ground by a MagNALyser in liquid nitrogen and the total antioxidants were extracted in 2 mL of ice cold 80% ethanol. FRAP (ferric reducing/antioxidant power assay) reagent (0.3 M acetate buffer (pH3.6), 0.01 mM TPTZ in 0.04 mM HCl, 0.02 M FeCl_3_.6H_2_O) was mixed in equal volume with the extract and measured at 600 nm using a microplate reader (Synergy Mx, Biotek Instruments Inc., Winooski, Vermont, USA) and trolox was used as standard [41].

### 2.12. Ascorbate, Glutathione, and Their Redox Status

Reduced ascorbate (ASC) and glutathione (GSH) were quantified by HPLC analysis according to Potters, Horemans [42]. A known concentration of reduced ascorbate and glutathione were used as standard for the HPLC run. Total ascorbate (tAsc) and total GSH (tGSH) was determined after reduction with 0.04 M dithiothreitol DTT for 10 min at room temperature, and the redox status was calculated as the ratio of the reduced form to the total concentration.

### 2.13. Tocopherols 

Tocopherols were extracted from 100 mg frozen tissues in 6 mL hexane using the MagNALyser and centrifuged at 14,000× *g* for 15 min. Extracts were dried (CentriVap concentrator, Labconco, Kansas City, MO, USA) and the dried extract (CentriVap concentrator, Labconco, KS, USA) was resuspended in hexane, and tocopherols were separated and quantified by HPLC (Shimadzu, ‘s Hertogenbosch, The Netherlands) (normal phase conditions, Particil Pac 5 µm column material, length 250 mm, i.d. 4.6 mm). Dimethyl tocol (DMT) was used as the internal standard (5 ppm). Data were analysed with Shimadzu Class VP 6.14 software. 

### 2.14. Polyphenols and Flavonoids 

Polyphenols and flavonoids were extracted by homogenizing 200 mg FW in 2 mL 80 percent ethanol (*v*/*v*), centrifugated and the clear supernatant was used to estimate polyphenols and flavonoids content. 

Polyphenols content was determined using a Folin–Ciocalteu assay according to [43]. Gallic acid was used as a standard. Flavonoid content was estimated using the modified aluminum chloride calorimetric method [44] with quercetin as a standard.

### 2.15. Enzyme Assays

#### 2.15.1. Photorespiration Enzymes

The hydroxypyruvate reductase (HPR) activity was estimated following Schwitzguebel and Siegenthaler [45]. The change in NADH extinction coefficient of 6.2 mM^−1^ cm^−1^ at 340 nm was used to follow the enzymatic activity. Glycolate oxidase activity (GO) was measured according to Feierabend and Beevers [46] by following the formation of glyoxylate complex with phenylhydrazine (ε_324_ = 17 mM^−1^ cm^−1^).

#### 2.15.2. Oxidative Stress-Related Enzymes

The activity of NADPH oxidases (NOX) was estimated according to [47]. The reduction rate of NBT into monoformazan (ε_530_ = 12.8 mM^−1^ cm^−1^) was used to assess NADPH-dependent superoxide generation.

#### 2.15.3. Antioxidant Enzymes 

For measurements of activities of ascorbate peroxidase (APX), dehydroascorbate reductase (DHAR), glutathione reductase (GR), peroxidase (POX), superoxide dismutase (SOD), and catalase (CAT), 100 mg of frozen shoot tissue was extracted (MagNALyser, 1 mL of extraction buffer: 50 mM MES/KOH (pH 6.0) and assayed in microplates following Murshed, Lopez-Lauri [48]. The oxidation of pyrogallol (ε_430_ = 2.47 mM^−1^ cm^−1^) was used to assess POX activity [49]. The method of Dhindsa, Plumb-Dhindsa [50] was used to calculate SOD activity by measuring the inhibition of NBT reduction at 560 nm. Catalase activity was calculated by measuring the rate of decomposition of H_2_O_2_ at 240 nm (ε_240_ = 43.6 mM^−1^ cm^−1^) as defined by Aebi [51]. Using a microplate reader (Synergy Mx, Biotek Instruments Inc., Winooski, Vermont, USA), all behavior measurements were scaled down for semi-high throughput and optimized to achieve linear time and protein-concentration dependence. The protein content in the enzyme extracts was determined using a Bradford assay (BioRad) [52].

### 2.16. Ascorbate Biosynthesis Enzymes

#### 2.16.1. L-Galactono-1,4-Lactone Dehydrogenase (GalLDH) Activity

Plant samples were homogenized in 1 mL of Tris-HCl buffer (0.1 M, pH 7.5) with sucrose (0.3 M), polyvinylpolypyrrolidone (1% *w*/*v*), BSA (0.2% (*w*/*v*), dithiothreitol (1 mM), and EDTA (50 mM). After centrifugation for 30 min at 35,000 rpm at 4 °C, the supernatants were used for enzyme assay. L-Galactono-1,4-Lactone Dehydrogenase (GalLDH) activity was assayed spectrophotometrically at by measuring the increase in A 550 accompanied by the reduction of cytochrome c as described by Ôba, Ishikawa [53]. 

#### 2.16.2. D-Galacturonic Acid Reductase (GalUR) Activity

Plant samples homogenized in liquid nitrogen were extracted in MOPS buffer (20 mM, pH 6.9) with MgCl_2_ (1 mM), b-mercaptoethanol (2 mM), EDTA (3 mM), EGTA (1 mM), glycerol (5%), sucrose (0.25 M) and protease inhibitor (1 mM PMSF). After centrifugation for 30 min at 35,000 rpm at 4 °C, the supernatants were used for enzyme assay. According to Agius, González-Lamothe [54], D-Galacturonic acid reductase (GalUR) activity was measured in phosphate buffer (50 mM, pH 7.2), EDTA (2 mM), NADPH (0.1 mM), D- galacturonic acid (30 mM) and DTT (2 mM). The activity was estimated by measuring the decrease in absorbance at 340 nm at room temperature. The GalUR activity was expressed as nmol of NADPH oxidized min/mg protein.

### 2.17. Statistical Analysis, Heat Map, Principal Component Analyses (PCA) and % of Change

One way analysis of difference (ANOVA) of data was performed using GraphPad Prism 9.0 software. Significant differences between the means of parameters were determined by using the Tukey post-hoc test (*p* < 0.05). Data were checked by a D’Agostino & Pearson omnibus normality test, a Brown-Forsythe test, and Bartlett’s test for equal variances. Data are presented as means of at least three biological replicates ± SE. Principal components analysis (PCA) was run on R software. PCA graphs were created showing the distribution of individual samples in the first two PCA dimensions. The parameters and the degree to which they contribute to the total variation explained by the first two PCA dimensions were depicted as arrows. Hierarchical clustering analysis was generated by Multi Experimental Viewer (TM4 software package). The percentage (%) of changes was calculated as ((Treatment-Control)/Control) × 100

## 3. Results

### 3.1. Soil Analysis 

Table 1 shows that soil amendment with mycorrhiza retained their vanadium content after plant growth compared to the soils implemented with vanadium only. Relative to their control counterparts, treatment with vanadium or vanadium combined with mycorrhiza greatly enhanced sorghum soil phenolic content (by 199% and 248%, respectively) and to a lesser extent in rye soil (by 136 and 194%, respectively). Moreover, citrate content was significantly increased in soils with sorghum exposed to any of the treatments, notably the combined vanadium and mycorrhizal treatment. Treatment with only mycorrhiza enhanced soil content of phenolic by 160% in case of rye and citrate by 230% in case of sorghum.

### 3.2. Root Colonization and Content of Phosphatases, Proline and Citrate

The soil colonization, hyphal length and number of arbuscules were significantly decreased when vanadium treatment was combined with mycorrhizal treatment compared to mycorrhizal treatment alone. The reduction in the three forementioned parameters (45%, 41%, 27% in rye and 20%, 22%, 19.6% in sorghum, respectively) were higher in case of rye compared to those observed in sorghum (Table 1).

The root activities of alkaline and acid phosphatase and content of proline and citrate were significantly improved in rye roots when soil was amended with mycorrhiza, while vanadium treatment did not make significant changes in their values compared to their respective controls (except for a reduction in acid phosphatase activity). However, when vanadium treatment was combined with mycorrhizal treatment, the values of the measured parameters restored their control values. A similar trend of changes in values of proline and citrate contents could be observed in sorghum roots; however, their alkaline and acid phosphatase activities responded differently compared to what was observed in rye roots. Mycorrhiza increased only the alkaline phosphatase in sorghum roots while vanadium treatment decreased only their acid phosphatase activity, but this reduction was restored to control values when vanadium treatment was combined with mycorrhizal treatment.

### 3.3. Minerals Content in Roots and Shoots

Vanadium treatment adversely affected the rye shoots and roots content of K, Ca, Mg and P. In sorghum tissues, only Ca and Mg contents were negatively affected by vanadium. Mycorrhizal treatment, on the other hand, significantly improved rye shoots K content, rye roots K, Ca, P and Mn contents as well as sorghum roots and shoots contents of K, P and Mn. Mycorrhizal treatment could partially restore the loss of mineral content due to vanadium’s effect only in the case of rye shoot P, rye root Mg and P, as well as sorghum roots Ca and Mg (Table 2).

### 3.4. Vanadium Accumulation

**Figure 1:**
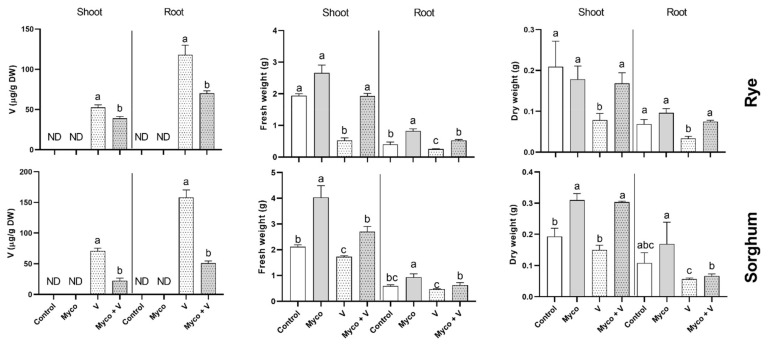
Accumulation of vanadium and changes in fresh and dry weights of shoots and roots in six-week-old rye and sorghum grown in soils with 0 or 350 mg/kg soil sodium vanadate and with or without soil enrichment with inoculum of the mycorrhiza *Rhizophagus irregularis*. Data are mean values ± SE (*n* = 3). Data were statistically analyzed by one-way ANOVA followed by Tukey’s posthoc test for comparing the means. Different letters indicate a statistically significant difference between means of the same plant species at a significance level of at least (*p* ≤ 0.05).

Both plant species accumulated Vanadium in their shoots and roots, however, higher accumulation can be observed in roots in both plants compared to shoots. Mycorrhizal treatment reduced vanadium accumulation in shoots (by 29% and 58% in rye and sorghum, respectively). A similar but more pronounced reduction in vanadium accumulation can be observed in roots (40% and 68% reduction in rye and sorghum, respectively) (Figure 1).

### 3.5. Growth

A sharp reduction in fresh and dry weight of rye shoots (74% and 62%, respectively) and roots (38% and 39%, respectively) grown in soil contaminated with vanadium (Figure 1) was noted. A less pronounced reduction in fresh and dry weights of sorghum shoots and roots was also observed. Mycorrhizal treatment fully restored fresh and dry weights of shoots and roots in both plant species. Growth of shoots and roots in rye were insensitive to mycorrhizal treatment while sorghum fresh weight of shoots and roots was remarkably enhanced (216% and 158%, respectively). The fresh weight of sorghum roots was slightly enhanced by mycorrhizal treatment, while its dry weight was not changed. 

### 3.6. Photosynthesis

**Figure 2:**
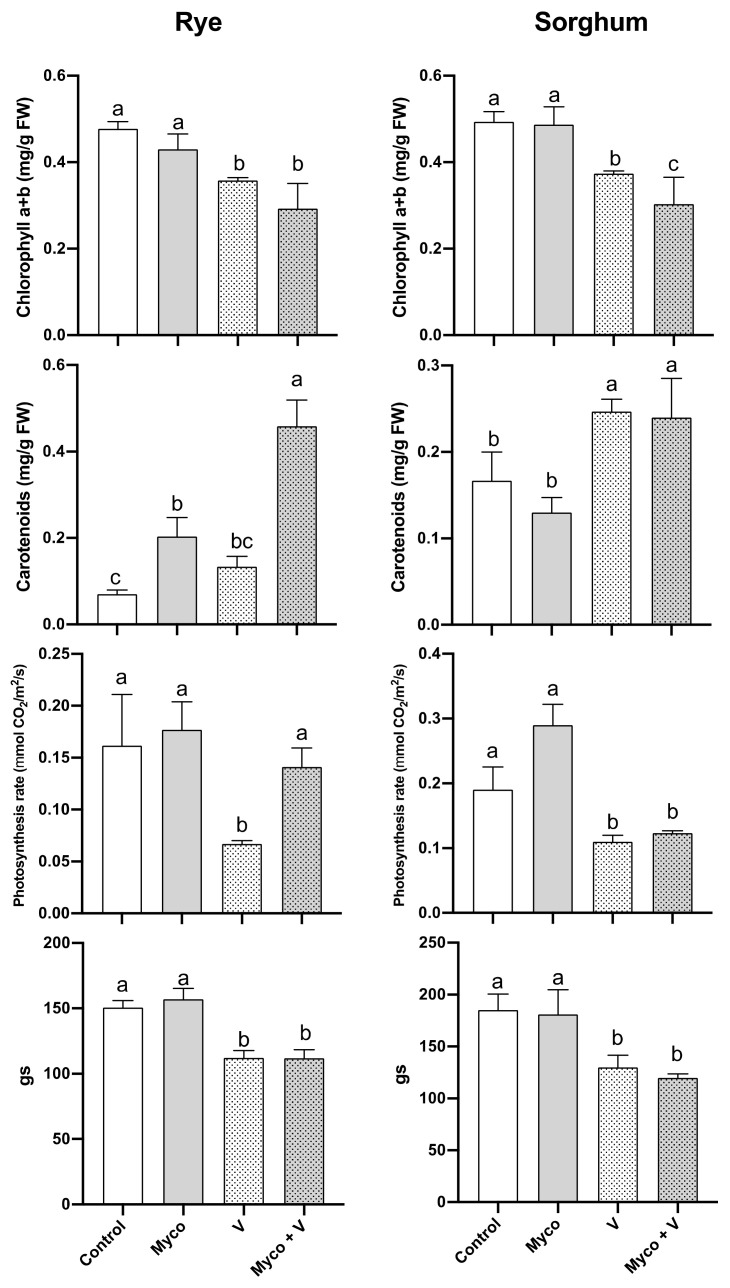
Changes in the photosynthetic parameters (total chlorophyll and carotenoid content, photosynthesis and gas exchange rates) of six-week-old rye and sorghum grown in soils with 0 or 350 mg/kg soil sodium vanadate and with or without soil enrichment with inoculum of the mycorrhiza *Rhizophagus irregularis*. Data are mean values ± SE (*n* = 3). Data were statistically analyzed by one-way ANOVA followed by Tukey’s posthoc test for comparing the means. Different letters indicate statistically significant difference between means of the same plant species at significance level of at least (*p* ≤ 0.05).

The total chlorophyll content was similarly reduced in rye and sorghum under vanadium stress (Figure 2) and hence a comparable reduction in photosynthetic rates (57% in rye and 41% in sorghum) can be observed. On the other hand, the carotenoids content was induced by vanadium treatment. Gas exchange rates (gs) were reduced by vanadium treatment. Except for the increase in photosynthesis of vanadium stressed rye and controlled sorghum plants, mycorrhizal treatment or when it was combined with vanadium did not affect any of the other measured parameters compared to control or vanadium treatment, respectively. 

### 3.7. The Sugars Metabolism

The changes in sugars and their metabolizing enzymes under mycorrhiza, vanadium and their combination are shown in Table 3. Mycorrhizal treatment improved the total soluble sugars, sucrose, glucose, fructose, insoluble sugars content and activities of sucrose-P-phosphate in shoots and roots as well as invertase and cell wall (CW) invertase in roots of rye and sorghum plants. The highest increase was in total soluble sugars and the increase was higher in rye shoots compared to roots (229% and 149% increase, respectively) while in sorghum roots increased content in total soluble sugars was higher compared to shoots (370% and 215% increase, respectively). Vanadium treatment, on the other hand, reduced the total soluble sugars, glucose, and insoluble sugars content while activities of sucrose-P-phosphate in shoots and roots of rye and sorghum and CW invertase in roots of both plants were boosted. Mycorrhizal treatment, combined with vanadium treatment, improved the measured sugars content and their metabolizing activities in shoots and roots of both plant species compared to their values under vanadium treatment.

### 3.8. Oxidative Stress and Photorespiration

**Figure 3:**
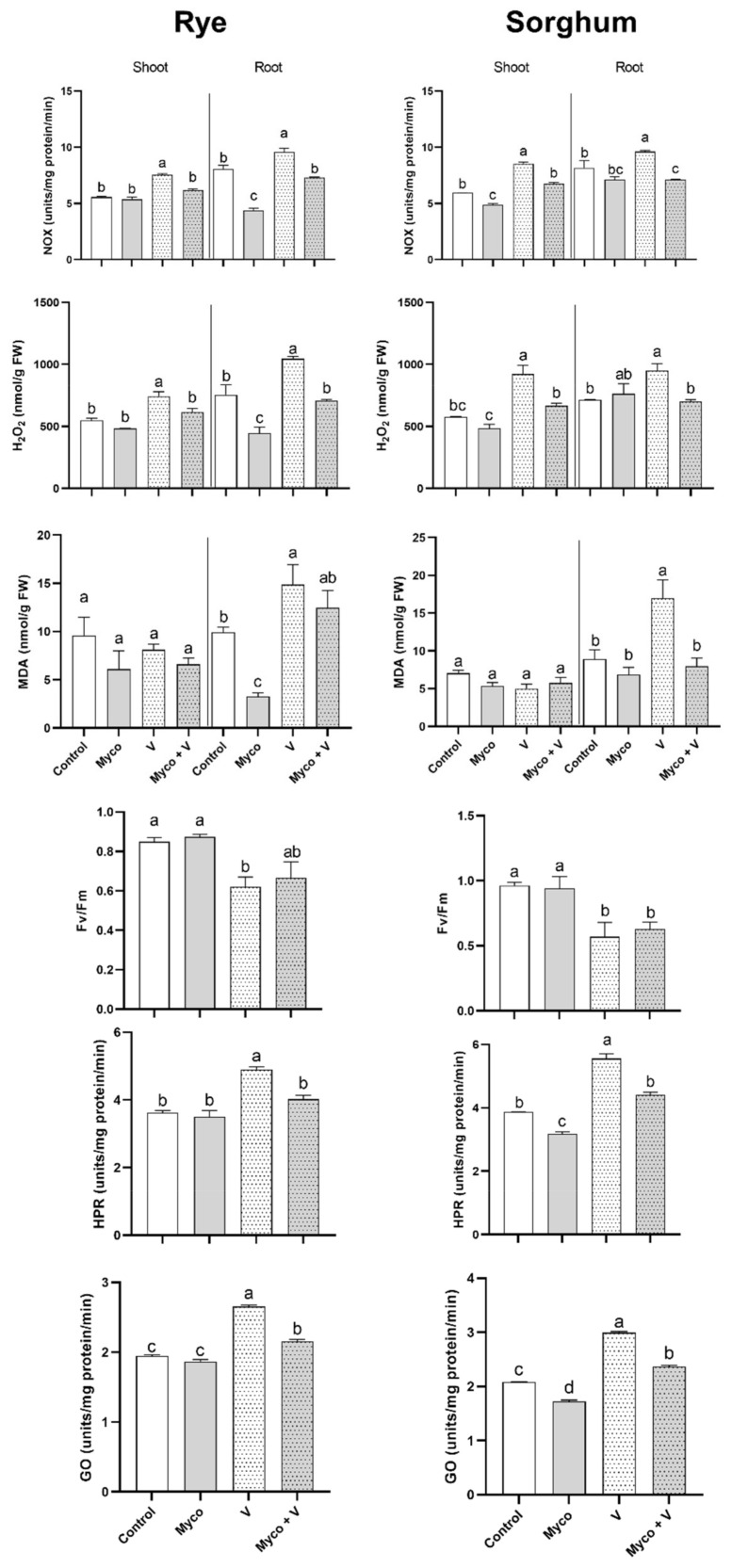
Changes in oxidative stress parameters (NOX, H_2_O_2_, and MDA) in shoots and roots and photorespiration enzymes (F_v_/F_m_ ratio, HPR and GO) in shoots of six-week-old rye and sorghum grown in soils with 0 or 350 mg/kg soil sodium vanadate and with or without soil enrichment with inoculum of the mycorrhiza *Rhizophagus irregularis*. Data are mean values ± SE (*n* = 3). Data were statistically analyzed by one-way ANOVA followed by Tukey’s posthoc test for comparing the means. Different letters indicate statistically significant difference between means of the same plant species at significance level of at least (*p* ≤ 0.05).

The change in oxidative stress parameters under vanadium, mycorrhizal treatments and their combination is shown in Figure 3. Vanadium treatment increased the NADPH oxidase (NOX) activity in shoots and roots of rye and sorghum as compared to their controls. This increase was more pronounced in shoots (by 135% and 155% in rye and sorghum, respectively) than roots (by 123% and 113% in rye and sorghum, respectively), respectively (Figure 3). Similarly, vanadium treatment induced accumulation of higher concentrations of H_2_O_2_ in shoots (135% and 159%) and roots (138% and 133%) of rye and sorghum, respectively compared to control plants. However, when vanadium was combined with AMF, no change was observed in H_2_O_2_ levels. Only in rye roots did AMF reduced their H_2_O_2_ content (Figure 3).

In both plants, the MDA content in roots was only increased due to vanadium treatment while no significant changes in shoots could be observed. AMF restored control MDA level in roots of both plants when combined with vanadium.

A 27% and 40% reduction in the quantum efficiency of photosystem II was observed in rye and sorghum under vanadium treatment, respectively and when accompanied by mycorrhizal treatment, F_v_/F_m_ ratio was partially recovered in rye but did not affect it in sorghum.

Since photorespiration is an important source of reactive oxygen species especially under stress conditions, the changes in two key enzymes of photorespiration, namely hydroxy pyruvate reductase (HPR) and glycolate oxidase (GO) show that the activities of both enzymes were enhanced (by 135% and 137% in rye and by 138% and 143% in sorghum, for HPR and GO, respectively) in plants exposed to vanadium treatment. This enhancement was significantly reduced to control levels when vanadium treatment was combined with AMF; however, AMF alone did not induce any change in activities of HPR and GO.

### 3.9. Heavy Metals Chelating Proteins

**Figure 4:**
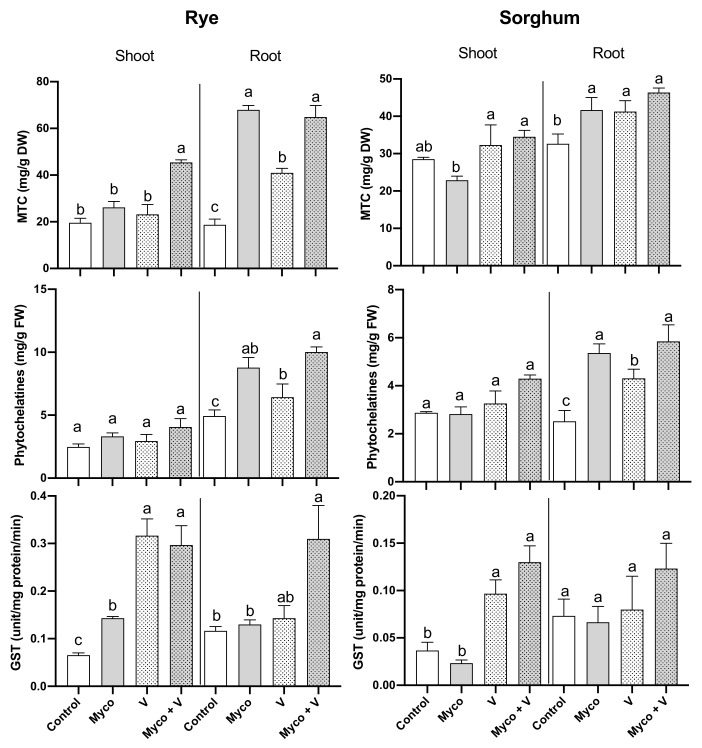
Changes in MTC and phytochelatins content and GST activity in shoots and roots of six-week-old rye and sorghum grown in soils with 0 or 350 mg/kg soil sodium vanadate and with or without soil enrichment with inoculum of the mycorrhiza *Rhizophagus irregularis*. Data are mean values ± SE (*n* = 3). Data were statistically analyzed by one-way ANOVA followed by Tukey’s posthoc test for comparing the means. Different letters indicate statistically significant difference between means of the same plant species at significance level of at least (*p* ≤ 0.05).

Figure 4 shows the changes in metallothioneins (MTC), phytochelatins (PC) and GST activity in roots and shoots of rye and sorghum plants exposed to mycorrhizal proteins, vanadium or their combination. MTC and PC contents in shoots of both plants were not changed when plants were exposed to vanadium treatment. However, the shoots activity of GST activity was enhanced due to vanadium treatment by 364% (in rye) and 272% (in sorghum) compared to their control counterparts. Sorghum roots contents of MTC and PC in sorghum were increased due to vanadium treatment. On the other hand, GST activity increased in sorghum shoots. MTC and PC content in rye roots was increased compared to its control. Under mycorrhizal treatment, some of measured parameters were changed in rye shoots, sorghum shoots, and roots compared to their control counterparts. Under AMF treatment, the MTC and PC contents in rye roots were increased by 310% and 160% relative to their controls, respectively. Similarly, MTC and PC contents were increased in sorghum roots. A combination of vanadium and mycorrhizal treatment improved the rye and sorghum shoots content of GST activity, the rye roots content of MTC, PC and GST activity, and sorghum root content of PC. 

### 3.10. Nonenzymatic Antioxidants

Supplementary Figure S1 shows that none of the measured parameters was changed in shoots and roots of sorghum under any of the treatments. In rye, an increase in phenolics content could be observed in shoots and roots of plants exposed to vanadium or vanadium+AMF plants compared to their control values, respectively. Moreover, a pronounced increase in total tocopherols (165%) and ASC/total ASC ratio (311%) in rye roots could be observed in plants grown in soils enriched with mycorrhiza. 

### 3.11. Enzymatic Antioxidants

**Figure 5:**
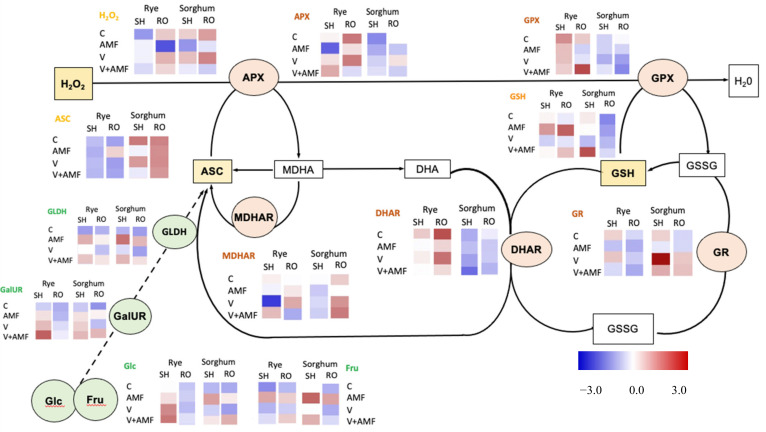
Changes in ascorbate-glutathione cycle enzymes and metabolites, glucose, fructose and two key enzymes in the metabolizing sugars into ascorbate in shoots and roots of six-week-old rye and sorghum grown in soils with 0 or 350 mg/kg soil sodium vanadate and with or without soil enrichment with inoculum of the mycorrhiza *Rhizophagus irregularis*.

Changes in ascorbate-glutathione enzymes and metabolites and two key enzymes in pathway of sugars metabolism into ascorbate are shown in Figure 5. Organ and species-specific changes could be observed in ascorbate-glutathione cycle enzymes and metabolites. APX activity was similarly enhanced in shoots of both plants exposed to vanadium or combined vanadium and mycorrhizal treatments. 

A species-specific change could be observed in GR activity and GSH content. When exposed to vanadium or vanadium+AMF treatment, shoots and roots of only sorghum showed improved GR activity relative to their respective controls. The content of GSH of only rye shoots and roots was significantly increased under AMF or V+AMF treatments. Asc content was significantly increased under AMF treatment in rye roots and sorghum shoots. No statistically significant changes could be observed for MDHAR nor DHAR activities in shoots nor roots of sorghum or rye plants. 

**Figure 6:**
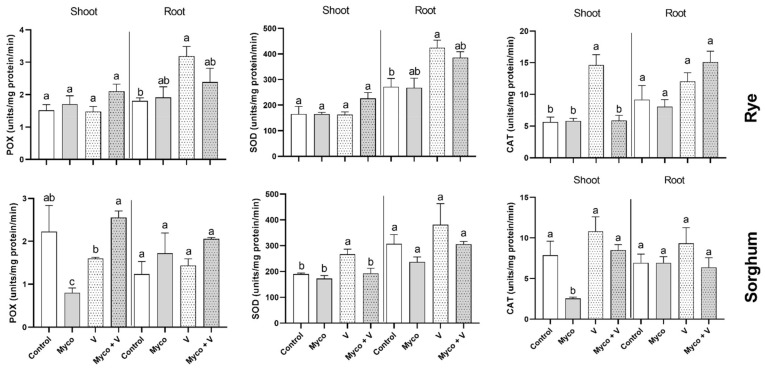
Changes in H_2_O_2_ scavenging enzymes (POX, SOD, and CAT) in shoots and roots of six-week-old rye and sorghum grown in soils with 0 or 350 mg/kg soil sodium vanadate and with or without soil enrichment with inoculum of the mycorrhiza *Rhizophagus irregularis*. Data are mean values ± SE (*n* = 3). Data were statistically analyzed by one-way ANOVA followed by Tukey posthoc test for comparing the means. Different letters indicate statistically significant difference between means of the same plant species at significance level of at least (*p* ≤ 0.05).

The changes in activities of direct H_2_O_2_ scavenging enzymes is shown in Figure 6. POX and SOD showed a remarkable increase in their activities (by 176% and 156%, respectively) in rye roots under vanadium treatment compared to their control values. When vanadium was combined with AMF, such increases in POX and SOD in rye roots were eliminated. When exposed to vanadium treatment rye shoots are distinguished with increased CAT activity (262%), while sorghum shoots showed improved GR activity (197%) relative to their respective controls.

### 3.12. Ascorbate Biosynthesis

To understand how AMF and V effect on Asc biosynthesis, we measured the activity of tow key enzymes involved in Asc biosynthesis i.e., L-Galactono-1,4-Lactone Dehydrogenase (GalLDH) and (GalUR). The GLDH activity was boosted by all treatments in shoots and roots of both plants. On the other hand, GalUR activity was enhanced by V, AMF and A+AMF treatments in shoots of rye and sorghum, while in their roots, its activity was significantly increased only after AMF and AMF+V treatments (Figure 5). 

### 3.13. Principal Components Analysis

**Figure 7:**
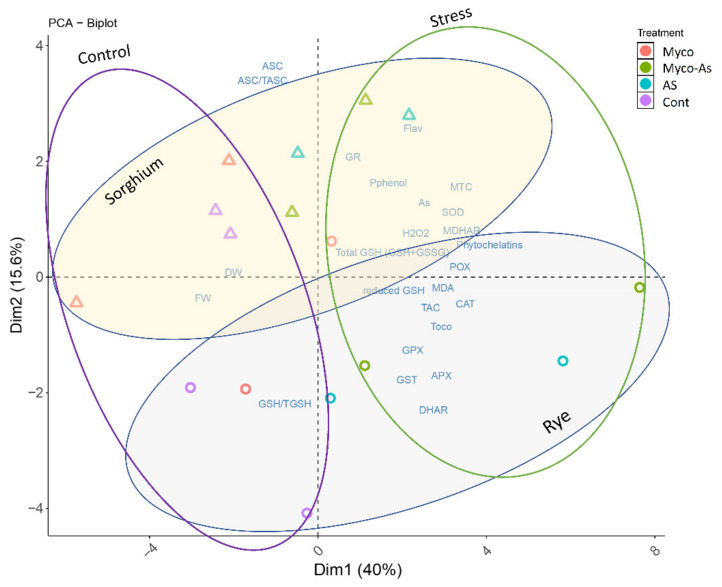
Principal component analysis (PCA) of vanadium accumulation, growth, MTC, PC, enzymatic and nonenzymatic antioxidants in shoots and roots of six-week-old rye and sorghum grown in soils with 0 or 350 mg/kg soil sodium vanadate and with or without soil enrichment with inoculum of the mycorrhiza *Rhizophagus irregularis*.

To test the specific responses of different plant and organs to vanadium accumulation and soil enrichment with AMF, principal component analysis (PCA) was run using all parameters were measured in shoots and roots of rye (C3) and sorghum (C4) (Figure 7). The biplot embodies uniform parameters along the first two PC that declares about 40% and 15% of the data variability, respectively. PC1 clearly separates the measured parameters on the base of vanadium stress condition (40%), while PC2 clearly separated the two plants (15%). Overall, the PCA demonstrated that antioxidant parameters are separated based on plant species and vanadium stress indicating their key role in the difference between the two plants. 

## 4. Discussion

The beneficial effect of vanadium on plant growth and productivity was reported; however, the essentiality of vanadium for plant’s life is controversial [55,56]. On the other hand, vanadium concentration in soil above a certain threshold is detrimental to plant growth [1]. Our study aimed to extend the progress in vanadium research made so far to understand the mechanism of vanadium accumulation and the extent of its phytotoxicity in shoots and roots of C3 an C4 plants, which may help in regulating vanadium tolerance in different plant species. Moreover, exploring the mechanism of vanadium-induced tolerance help developing plants capable of tolerating high vanadium concentration and producing sufficient yields. Here the manuscript reported the absorption and accumulation of vanadium by rye and sorghum plants.

Our results indicate a higher accumulation of V in roots of both plants compared to their shoots and higher accumulation in sorghum roots compared to their rye counterparts. This is consistent with previous studies that showed higher retention of vanadium in root tissues rather than its translocation to the shoot system in tomato [9], pepper [8] and rice [10]. These differences in the degree of vanadium accumulation in roots and shoots might lower the translocation from root to shoot [57]. 

Vanadium can be taken up by plants in place of phosphorus [1], thus the impact of AMF on phosphorus uptake was investigated. AMF can mineralize and improve the acquisition of organic phosphorus by inducing phosphatase activities in AMF treated plant roots [58]. Given the important role of root phosphatase in organic phosphorus sources bioavailability, root acid and alkaline phosphatase activities in roots of both plants were investigated. Similar to our results, AMF increased acid and alkaline phosphatase activity in colonized soybean roots [59,60]. In this regard, Fries, Pacovsky [60] reported that AMF increased acid phosphatase activity due to increased inorganic phosphorus release from phosphorus mineralization, leading to an increase in the availability of nutrients. Overall, AMF reduced V accumulation and increased mineral availability could explain the improvement in biomass for both plants under arsenic stress. In agreement with our findings, Amaya-Carpio, Davies [61] reported that treatment of *Ipomoea carnea* with AMF increased nutrient acquisition from an organic fertilizer source by enhancing the activity of acid and alkaline phosphatase which facilitated phosphorus acquisition, increasing photosynthesis and improving plant growth.

An earlier study indicated that vanadium inhibited growth of human cells by arresting G2/M cell cycle through ROS-mediated reactions [62]. The results suggest that the vanadium accumulation resulted in reduced growth at fresh and dry weights of the shoot and root systems in both plant species. However, the retardation of growth in shoots that accumulated less vanadium is higher than roots that accumulated higher concentrations of vanadium, indicating a higher sensitivity of shoot metabolism to vanadium. This could be ascribed, at least partially, to the reduction in chlorophyll content and photosynthetic efficiency. Similarly, shoots of rice seedling were more sensitive to vanadium compared to roots, as indicated by reduced length, fresh and dry weights and its lower content of chlorophyll, and their photosynthesis were largely reduced [10]. Vanadium greatly inhibited chlorophyll biosynthesis and interfered with sulfur-containing amino acids, which resulted in decreased plant metabolism [57]. 

Moreover, rye and sorghum experienced different sensitivities towards vanadium, rye was being more sensitive, since it accumulated less vanadium than sorghum; however, it showed a larger reduction in fresh and dry weights. Furthermore, rye exhibited a reduction in gas exchange and a stronger inhibition of photosynthesis than sorghum. These differences in the response of photosynthesis of the two plants could be at least partly attributed to their difference in the type of photosynthesis [63]. C4 plants like sorghum concentrate CO_2_ around the carbon fixing cells and do minimal photorespiration, hence less ROS generation and their photosynthetic machinery was more efficient and less labile to stomatal closure and adverse conditions compared to C3 plants [64,65]. The reduction in chlorophyll content and hence photosynthetic rate and accumulation of dry matter could be attributed to vanadium interference with root absorption and tissue content of Mg, as our results show. Mg is a structural component essential for the functionality of photosynthetic machinery [66]. In support to our findings, rice seedlings exposed to increasing concentrations of vanadium show reduction in their chlorophyll content and photosynthetic rate in a vanadium-concentration dependent manner [10]. Moreover, Olness, Palmquist [67] showed that vanadium interfered with the absorption of magnesium and calcium by soybean roots.

The larger reduction in growth and the sensitivity of rye compared to sorghum towards vanadium could also be due to vanadium’s interference with four essential nutrients in rye (K, Ca, Mg and P) compared to the reduction in only Ca and Mg in sorghum shoots and roots. In this regard, vanadium inhibits the plasma membrane hydrogen-translocation ATPase [68], which involves nutrient uptake in plants. Vanadium also reduced the activity of the divalent cation transporter (ZIP) [55], and interfered with the activity of phosphatases, ribonucleases and protein kinases, while activating NADPH oxidase and inducing genotoxicity effects [69]. For instance, it decreased the P concentrations in soybean plants [70]. AMF are one of the most important soil microorganisms that develop mutual symbiotic association with plant roots [16]. Mycorrhizal treatment improved more nutrient (K, P and Mn) accumulation in sorghum shoots compared to improved content of only K in sorghum shoots. Similarly, AMF facilitated the uptake and transport of phosphorus and other relatively immobile soil nutrients, promoted plant growth and enhanced their stress tolerance [23]. Furthermore, mycorrhizal treatment fully or at least partially restored the reduction of Ca and Mg content in sorghum roots and hence restored growth in both plants and the photosynthetic rate in rye. The restoration of mineral content could be a result of increased area of contact with soil and to the secretion of citrate (in case of sorghum) which in turn lowered soil pH and facilitated mineral absorption. Similarly, Gashgari, Selim [18] reported the improvement in mineral content in parsley and pennyroyal grasses when grown in soils enriched with mycorrhiza.

Increased photosynthesis from increased sugar metabolism could allocate more sugars to plant roots that increase their sink strength [71]. Moreover, AMF colonization increased expression of source-to-sink metabolizing genes and transport of sugars [72]. Overall, the increased sugar levels were correlated with increased metabolism in shoot and root including sucrose P synthase and invertases. High sugars accumulation by AMF and V stress conditions may act as supplier of C skeletons for producing organic acids which in turn increased organic acid (citric acid) exudation into the soil to reduce V uptake. The excretion of organic acids by root form soluble complexes and chelates with metal ions and they could modify the fixation and mobility of heavy metals in soils [73]. In this regard, citric acid has been used as a viable environmental technology for the mobilizing of heavy metals [73].

Induced sugars accumulation in the plant root could also stimulate respiration which in turn increased intermediates levels such as the amino acid proline. The accumulation of soluble sugars and proline in response to AMF may also act as a strategy to regulate the osmotic status of stressed plants [74]. In this regard, the accumulated sugars play a pleiotropic role in plant physiology and metabolism, where they reserve as an energy source and organic building blocks as well as they can being involved in maintaining the osmotic potential of the plant cell, protecting membranes and stabilizing the photosystem II [75]. In addition to their osmoregulation potential, sugars have a high ROS scavenging capacity [76]. Furthermore, the high soluble sugars metabolism and accumulation in response to AMF under V stress conditions could have induced antioxidants, which play an important role in V stress mitigation. In this context, sugars can feed the NADPH- producing metabolism such as OPP pathway and thereby contribute to ROS scavenging [77]. UDP-glucose can be a precursor for carotenoid and ASC biosynthesis [78], as well as synthesis of the building blocks of GSH [79]. The ascorbate biosynthesis pathway through the D-galacturonic acid has been discovered in certain plant tissues and it represents a sugars salvage mechanism [54]. Similarly, the results showed that the key ascorbate biosynthetic enzymes, GalUR and GLDH, were enhanced in rye and sorghum in tissue and in a treatment-specific manner. While GalUR activity showed a shoot specific enhancement under vanadium, AMF or their combination, GLDH activity increased in shoots and roots of rye and sorghum specifically under AMF or V+AMF treatments. This indicates that ascorbate plays a dual role under vanadium stress. On one hand it acts as an ROS scavenger, and on the other its biosynthesis is a mechanism for allocation of the accumulated sugars. 

Vanadium interacts with several proteins and affects numerous biological mechanisms, such as membrane-bound transport systems and lipid peroxidation [80]. Both plants experienced oxidative stress as indicated by increased MDA level in their roots and reduced quantum efficiency of photosystem II (reduced Fv/Fm value) as a result of vanadium treatment. Similarly, rice seedlings exposed to vanadium stress showed increases in oxidative stress markers like H_2_O_2_, MDA, electrolyte leakage, and reduction in quantum efficiency of photosystem II [10].Chickpea plants also substantially increased the H_2_O_2_ and MDA content [13,57]. Likewise, increased H_2_O_2_ and MDA levels in plant tissues was correlated with enhancement of the transcript abundance of respiratory burst oxidase genes. In addition, vanadium activated haloperoxidase gene expression plant tissue [56] that altogether caused severe oxidative damages. Thus, under vanadium stress, oxidative damages are triggered, leading to ROS generation, which induces the biomacromolecule damages. Consequently, the cell death and ion leakage were also positively increased. 

The two plants used a different antioxidants arsenal to combat the oxidative stress. In the study of Lin, Trinh [55], several network genes such as peroxidases, MDHAR, GR, glutaredoxin, thioredoxin and GST, were induced by vanadium stress in rice. In the line, rye exposed to vanadium stress enhanced its phenolic content in roots and shoots while CAT and APX activities were only enhanced in shoots and SOD in roots. Roychoudhury [1] stated that vanadium highly activates the antioxidative enzymes such as SOD, CAT and POX activity where CAT activity enabled elimination of H_2_O_2_ and POX protected cellular membranes against oxidative damages. Vanadium increased the antioxidant enzyme activities probably due to the generations of additional levels of ROS that result from increased oxidative damages [1]. Sorghum, on the other hand, is dependent on the ascorbate-glutathione cycle enzymes APX and GR and activities to reduce the vanadium-induced oxidative stress. In accordance with the results, SOD, CAT and POX activities were increased in response to vanadium treatment in a concentration-dependent manner [10]. The observed enhancement of CAT and APX activities in rye but not sorghum due to vanadium stress could be a reflection of the induced oxidative stress that could be associated with its type of photosynthesis (C3) compared to sorghum (C4 plant). 

To overcome vanadium toxicity, plant tissues stimulate the biosynthesis of metal chelators like MTC and PC as well as the metal detoxifying enzyme GST [1,81]. Both rye and sorghum increased their contents of GST activity in their shoots, while only sorghum accumulated MTC in its roots, reflecting the difference in their sensitivity towards vanadium. 

The results showed clear differences in the response of rye (C3) and sorghum (C4) towards vanadium stress, and mycorrhizal treatments mainly improved the mineral nutrients absorption and hence ameliorated the injurious effects of vanadium on key metabolites. The difference in response of the two plants are, at least partly, due to the differences in the type of photosynthesis. The importance of studies like this is to highlight metabolic pathways that are specific for certain plant species or groups that are upregulated under certain stress factors so that they can be manipulated either genetically or by using chemical or biological factors to have crops that are specifically capable of coping with these stress factors.

## 5. Conclusions

Overall, the current study emphasizes on the importance of investigating the specificity of plant organs/species/groups in their response to specific stress factors. Our work shows that the beneficial effect of AMF in improving the negative impact of soil V on roots and shoots growth was linked to their potentiality to improve plant photosynthesis (hence sugar content), which in turn provided energy and carbon backbone for scavenging the V-stress induced ROS accumulation. Moreover, AMF reduced V uptake in both plants to different degrees. Rye and sorghum used different ROS scavengers in an organ- and species-specific manner reflecting differences between the C3 and C4 metabolism types. These results, along with results obtained from similar studies, will enable researchers to synthesize overall conclusions about the differences between C3 and C4 plants that may be understood in their evolutionary context. Moreover, this work draws more attention to the efficiency of using environment-friendly methods to alleviate injurious stress effects and to improve crop production.

## Figures and Tables

**Table 1:** Changes in soil vanadium (mg/Kg), phenolic and citrate content after harvesting and alkaline and acid phosphatases, proline and citrate contents, mycorrhizal colonization %, hyphal length and number of arbuscules of six-week-old rye and sorghum shoots and roots grown in soils with 0 or 350 mg/kg soil sodium vanadate and with or without soil enrichment with inoculum of the mycorrhiza *Rhizophagus irregularis*. nd means not detectable. Data are mean values ± SE (*n* = 3). Data were statistically analyzed by one-way ANOVA followed by Tukey’s posthoc test for comparing the means.

	Treatment	V Content	Phenolic Content	Citrate Content	Alkaline Phosphatase	Acid Phosphatase	Proline Content	Citrate Content	Mycorrhizal Colonization (%)	Hyphal Length (cm.g-1 Soil)	No. Arbuscules (No. cm-1 Root)
Rye	Control	nd	74.8 ± 0.8 ^b^	10.9 ± 1.2 ^a^	3.1 ± 0.3 ^a,b^	5.6 ± 0.1 ^b^	4.3 ± 0.8 ^a^	2.4 ± 0.17 ^a^	0 ± 0 ^a^	0 ± 0 ^a^	0 ± 0 ^a^
	Myco	nd	118 ± 15 ^a^	6.94 ± 0.2 ^a^	5.5 ± 0.7 ^d^	8 ± 0.9 ^c^	7.6 ± 0.1 ^c^	4.8 ± 0.31 ^b^	55.3 ± 3.7 ^c^	18.3 ± 0.9 ^c^	4.4 ± 0.01 ^b^
	V	77 ± 1.3 ^b^	101.9 ± 3 ^a^	7.05 ± 0.6 ^a^	2.4 ± 0.4 ^a^	3.5 ± 0.5 ^a^	4.7 ± 0.5 ^a^	3.1 ± 0.22 ^a^	0 ± 0 ^a^	0 ± 0 ^a^	0 ± 0 ^a^
	Myco + V	98 ± 5.4 ^a^	145 ± 28 ^a^	11.8 ± 1.6 ^a^	3.9 ± 0.2 ^c^	5.6 ± 0.7 ^b^	5.8 ± 0.9 ^b^	5.8 ± 0.26 ^b^	30.3 ± 1.8 ^b^	10.8 ± 1.75 ^b^	3.2 ± 0.83 ^b^
Sorghium	Control	nd	75.12 ± 9 ^b^	2.76 ± 0.1 ^c^	4 ± 0.53 ^a^	7.5 ± 0.4 ^b^	6.6 ± 0.4 ^a^	2.4 ± 0.16 ^a^	0 ± 0 ^a^	0 ± 0 ^a^	0 ± 0 ^a^
	Myco	nd	79 ± 7 ^b^	6.19 ± 0.7 ^b^	8.9 ± 0.1 ^b^	9.6 ± 0.5 ^b^	9.7 ± 0.7 b	5.4 ± 0.3 ^c^	60 ± 2.6 ^c^	24.9 ± 0.5 ^c^	6.1 ± 0.5 ^c^
	V	77 ± 5.2 ^a^	149 ± 10 ^a^	7.12 ± 0.5 ^b^	3 ± 0.66 ^a^	5.1 ± 0.5 ^a^	6 ± 0.5 ^a^	3.6 ± 0.24 ^b^	0 ± 0 ^a^	0 ± 0 ^a^	0 ± 0 ^a^
	Myco + V	108 ± 7 ^b^	186 ± 13 ^a^	15.38 ± 1 ^a^	4.9 ± 0.4 ^a^	8.3 ± 0.2 ^b^	8.1 ± 0.3 ^b^	7.7 ± 0.14 ^d^	47.9 ± 2.7 ^b^	19.3 ± 1.4 ^b^	4.9 ± 0.6 ^b^

^a, b, c, d^ Different letters indicate statistically significant difference between means of the same plant species at a significance level of at least (*p* ≤ 0.05). “nd” means not detectable.

**Table 2:** Changes in minerals content of six-week-old rye and sorghum shoots and roots grown in soils with 0 or 350 mg/kg soil sodium vanadate and with or without soil enrichment with inoculum of the mycorrhiza *Rhizophagus irregularis*. nd means not detectable. Data are mean values ± SE (*n* = 3). Data were statistically analyzed by one-way ANOVA followed by Tukey’s posthoc test for comparing the means.

	Organ	Treatment	K	Ca	Mg	P	Mn	Zn
Rye	Shoot	Control	2.38 ± 0.13 ^b^	0.50 ± 0.02 ^a^	0.55 ± 0.02 ^a^	3.46 ± 0.21 ^a,b^	0.18 ± 0.01 ^a,b^	0.07 ± 0.002 ^a^
		Myco	3.03 ± 0.25 ^a^	0.46 ± 0.04 ^a^	0.47 ± 0.04 ^a,b^	4.10 ± 0.34 ^a^	0.20 ± 0.02 ^a^	0.04 ± 0.003 ^a^
		V	0.67 ± 0.09 ^c^	0.34 ± 0.02 ^b^	0.41 ± 0.02 ^b^	1.30 ± 0.11 ^c^	0.14 ± 0.01 ^b,c^	0.05 ± 0.001 ^a^
		Myco + V	2.33 ± 0.07 ^b^	0.32 ± 0.04 ^b^	0.29 ± 0.02 ^c^	3.02 ± 0.07 ^b^	0.16 ± 0.002 ^b,c^	0.03 ± 0.003 ^b^
	Root	Control	2.29 ± 0.45 ^b^	0.27 ± 0.09 ^b^	0.46 ± 0.02 ^a^	2.90 ± 0.61 ^b^	0.15 ± 0.03 ^b,c^	0.02 ± 0.01 ^a^
		Myco	4.59 ± 0.29 ^a^	0.48 ± 0.05 ^a^	0.57 ± 0.04 ^a^	5.11 ± 0.32 ^a^	0.31 ± 0.02 ^a^	0.03 ± 0.003 ^a^
		V	1.42 ± 0.08 ^c^	0.17 ± 0.02 ^c^	0.11 ± 0.04 ^b^	1.58 ± 0.10 ^c^	0.13 ± 0.03 ^c^	0.02 ± 0.005 ^a^
		Myco + V	2.97 ± 0.1 ^b^	0.37 ± 0.02 ^a^	0.25 ± 0.03 ^c^	3.31 ± 0.17 ^b^	0.21 ± 0.01 ^b^	0.02 ± 0.001 ^a^
Sorghium	Shoot	Control	2.28 ± 0.26 ^b^	0.47 ± 0.05 ^a^	0.55 ± 0.03 ^a^	3.38 ± 0.25 ^b^	0.17 ± 0.01 ^c^	0.06 ± 0.004 ^a^
		Myco	4.81 ± 0.31 ^a^	0.57 ± 0.04 ^a^	0.54 ± 0.05 ^a^	6.20 ± 0.41 ^a^	0.33 ± 0.02 ^a^	0.07 ± 0.004 ^a^
		V	2.08 ± 0.06 ^b^	0.36 ± 0.0 ^b^	0.41 ± 0.01 ^b^	2.95 ± 0.06 ^b^	0.14 ± 0.01 ^c^	0.05 ± 0.003 ^a^
		Myco + V	3.34 ± 0.21 ^c^	0.53 ± 0.0 ^a^	0.33 ± 0.01 ^b^	4.21 ± 0.25 ^b^	0.23 ± 0.01 ^b^	0.05 ± 0.002 ^a^
	Root	Control	3.49 ± 0.38 ^b^	0.38 ± 0.03 ^a^	0.46 ± 0.01 ^a,b^	3.89 ± 0.42 ^b^	0.25 ± 0.03 ^b^	0.03 ± 0.01 ^a^
		Myco	5.51 ± 0.78 ^a^	0.50 ± 0.07 ^a^	0.60 ± 0.05 ^a^	6.12 ± 0.16 ^a^	0.45 ± 0.02 ^a^	0.05 ± 0.02 ^a^
		V	2.65 ± 0.08 ^b^	0.29 ± 0.02 ^b^	0.11 ± 0.09 ^c^	2.95 ± 0.09 ^b^	0.18 ± 0.01 ^b^	0.02 ± 0.001 ^a^
		Myco + V	3.47 ± 0.46 ^a,b^	0.34 ± 0.03 ^a,b^	0.32 ± 0.04 ^b^	3.85 ± 0.51 ^b^	0.24 ± 0.03 ^b^	0.02 ± 0.002 ^a^

^a, b, c^ Different letters indicate statistically significant difference between means of the same plant species at a significance level of at least (*p* ≤ 0.05). “nd” means not detectable.

**Table 3:** Changes in total soluble sugars, sucrose, glucose, fructose, insoluble sugars as well as the sugars metabolizing enzymes sucrose -P-synthase, invertase and cell wall (CW) invertase of six-week old rye and sorghum shoots and roots grown in soils with 0 or 350 mg/kg soil sodium vanadate and with or without soil enrichment with inoculum of the mycorrhiza *Rhizophagus irregularis*. Data are mean values ± SE (*n* = 3). Data were statistically analyzed by one-way ANOVA followed by Tukey’s posthoc test for comparing the means.

	Organ	Treatment	Soluble Sugars	Sucrose	Glucose	Fractose	Insoluble Sugars	Sucrose P Synthase	Invertase	CW Invertase
Rye	Shoot	Control	14.8 ± 2.1 ^c^	5.4 ± 0.7 ^c^	2.4 ± 0.2 ^b^	2.4 ± 0.3 ^d^	213 ± 19 ^b^	0.6 ± 0.06 ^d^	12.4 ± 0.1 ^c^	
		Myco	33.9 ± 1.24 ^a^	12.3 ± 1.1 ^a^	3.4 ± 0.8 ^b^	5.3 ± 0.4 ^a^	240 ± 17 ^a^	1.4 ± 0.1 ^b^	10 ± 0.04 ^d^	
		V	20.8 ± 4.22 ^b^	7.4 ± 1.8 ^b^	5.2 ± 0.3 ^a^	3.2 ± 0.03 ^c^	169 ± 6 ^c^	0.8 ± 0.07 ^c^	14.7 ± 0.9 ^a^	
		Myco + V	26.3 ± 2.1 ^b^	13.7 ± 0.5 ^a^	3.4 ± 0.1 ^b^	4.2 ± 0.02 ^b^	215 ± 19 ^b^	1.6 ± 0.1 ^a^	11.8 ± 0.1 ^b^	
	Root	Control	14.1 ± 1.0 ^c^	4.1 ± 0.32 ^b^	1.5 ± 0.12 ^c^	2.3 ± 0.1 ^d^	143.3 ± 8 ^c^	0.6 ± 0.05 ^c^	1.3 ± 0.08 ^b^	6.8 ± 0.7 ^c^
		Myco	21 ± 1.07 ^a^	7.7 ± 0.9 ^a^	2.4 ± 0.15 ^b^	4.9 ± 0.3 ^b^	204 ± 12 ^a^	1.6 ± 0.11 ^b^	2.3 ± 0.17 ^a^	14.2 ± 1.05 ^b^
		V	14.5 ± 1.1 ^c^	5.2 ± 0.4 ^a,b^	2.2 ± 0.12 ^b^	3.6 ± 0.3 ^c^	110.6 ± 9 ^d^	0.8 ± 0.07 ^c^	1.1 ± 0.11 ^b^	11.5 ± 0.8 ^b^
		Myco + V	18.6 ± 1.03 ^b^	6.8 ± 0.3 ^a^	3.7 ± 0.1 ^a^	6.2 ± 0.3 ^a^	171.8 ± 5 ^b^	1.9 ± 0.05 ^a^	2.6 ± 0.08 ^a^	19.5 ± 1.3 ^a^
Sorghium	Shoot	Control	22.7 ± 1.4 ^c^	9.2 ± 1.1 ^d^	2.3 ± 0.8 ^c^	4.1 ± 0.4 ^b^	195.9 ± 2 ^b^	0.5 ± 0.01 ^c^	11 ± 0.12 ^c^	
		Myco	48.8 ± 2.4 ^a^	14.1 ± 0.9 ^b^	4.8 ± 0.3 ^a^	7.7 ± 0.5 ^a^	226.3 ± 12 ^a^	1.4 ± 0.01 ^b^	15.6 ± 0.14 ^b^	
		V	29.7 ± 0.9 ^a,b^	12.6 ± 0.1 ^c^	2.9 ± 0.1 ^c^	4.7 ± 0.7 ^a,b^	157.3 ± 7 ^c^	0.9 ± 0.4 ^c^	16.8 ± 0.13 ^b^	
		Myco + V	38.3 ± 3.15 ^b^	18.5 ± 2 ^a^	4 ± 0.5 ^b^	6.2 ± 0.1 ^a^	190 ± 11 ^b^	1.9 ± 0.1 ^a^	19.7 ± 0.8 ^a^	
	Root	Control	10.9 ± 1 ^d^	5.2 ± 0.3 ^c^	1.5 ± 0.1 ^d^	2.4 ± 0.1 ^b^	157.7 ± 7 ^c^	0.8 ± 0.04 ^b^	1.3 ± 0.09 ^c^	12.8 ± 0.7 ^c^
		Myco	37.2 ± 2 ^a^	10.5 ± 0.6 ^a^	3.2 ± 0.2 ^b^	5.5 ± 0.9 ^a^	226.9 ± 13 ^a^	1.6 ± 0.08 ^a^	2.5 ± 0.1 ^a^	18.2 ± 0.9 ^a^
		V	14.2 ± 1 ^c^	7.2 ± 0.4 ^b^	2 ± 0.14 ^c^	3.2 ± 0.2 ^a,b^	115 ± 11 ^d^	0.7 ± 0.07 ^b^	1.7 ± 0.13 ^b^	14.1 ± 1 ^b,c^
		Myco + V	26.7 ± 1 ^b^	9.7 ± 0.3 ^a^	4.5 ± 0.1 ^a^	4.2 ± 0.1 ^a^	184.2 ± 5 ^b^	1.4 ± 0.01 ^a^	2.8 ± 0.0 ^a^	16.2 ± 0.5 ^b^

^a, b, c, d^ Different letters indicate statistically significant difference between means of the same plant species at significance level of at least (*p* ≤ 0.05).

## Data Availability

Data presented in this study are available on reasonable request.

## Supplementary Materials

The following are available online at https://www.mdpi.com/article/10.3390/jof7110915/s1, Figure S1: Changes in nonenzymatic antioxidants (phenolics, flavonoids, total tocopherols content, total antioxidant capacity (TAC), Asc/total Asc and GST/tital GST ratio) in shoots and roots of 6- weeks old rye and sorghum grown in soils with 0 or 350 mg/kg soil sodium vanadate and with or without soil enrichment with inoculum of the mycorrhiza Rhizophagus irregularis. Data are mean values ± SE (*n* = 3). Data were statistically analyzed by one-way ANOVA followed by Tukey posthoc test for comparing the means. Different letters indicate statistically significant difference between means of the same plant species at significance level at least (*p* ≤ 0.05). Figure S2: Changes in the two key enzymes, GLDH and GalUR, involved in ascorbate metabolism in shoots and roots of 6-weeks old rye and sorghum grown in soils with 0 or 350 mg/kg soil sodium vanadate and with or without soil enrichment with inoculum of the mycorrhiza Rhizophagus irregularis. Data are mean values ± SE (*n* = 3). Data were statistically analyzed by one-way ANOVA followed by Tukey posthoc test for comparing the means. Different letters indicate statistically significant difference between means of the same plant species at significance level at least (*p* ≤ 0.05).
