# Supplementary material for: Improved Mineral Acquisition, Sugars Metabolism and Redox Status after Mycorrhizal Inoculation Are the Basis for Tolerance to Vanadium Stress in C3 and C4 Grasses
Source: J Fungi (Basel). 2021 Oct 27;7(11):915. doi: 10.3390/jof7110915 (PMC8625288; doi:10.3390/jof7110915)
Supplement: Supplementary file 1 [file jof-07-00915-s001.zip › jof-1405549-supplementary.pdf]

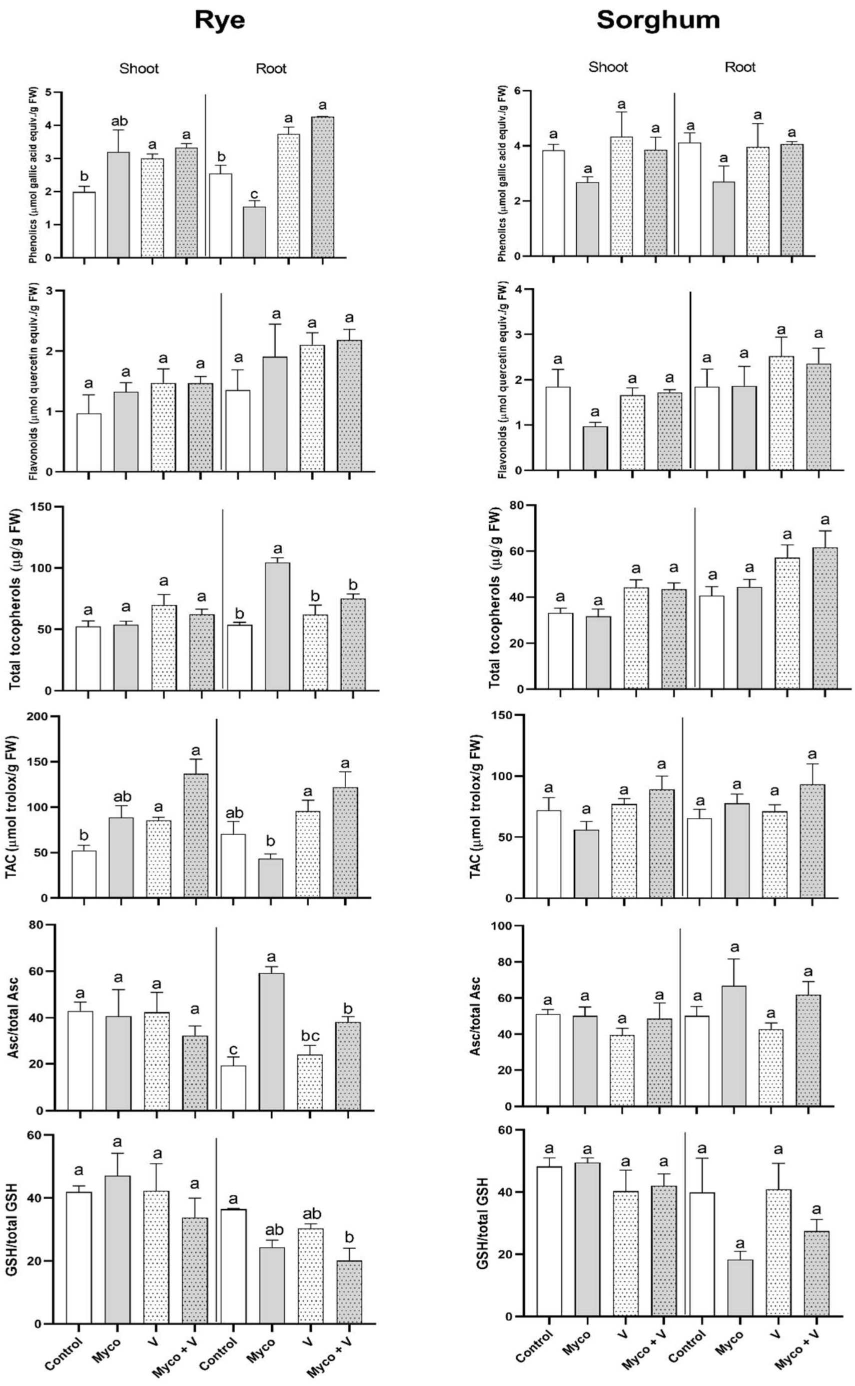

Supplementary Figure S1: Changes in nonenzymatic antioxidants (phenolics, flavonoids, total tocopherols content, total antioxidant capacity (TAC), Asc/total Asc and GST/tital GST ratio) in shoots and roots of 6-weeks old rye and sorghum grown in soils with 0 or 350 mg/kg soil sodium vanadate and with or without soil enrichment with inoculum of the mycorrhiza *Rhizophagus irregularis*. Data are mean values ± SE (n=3). Data were statistically analyzed by one-way ANOVA followed by Tukey posthoc test for comparing the means. Different letters indicate statistically significant difference between means of the same plant species at significance level at least ($P \leq 0.05$).

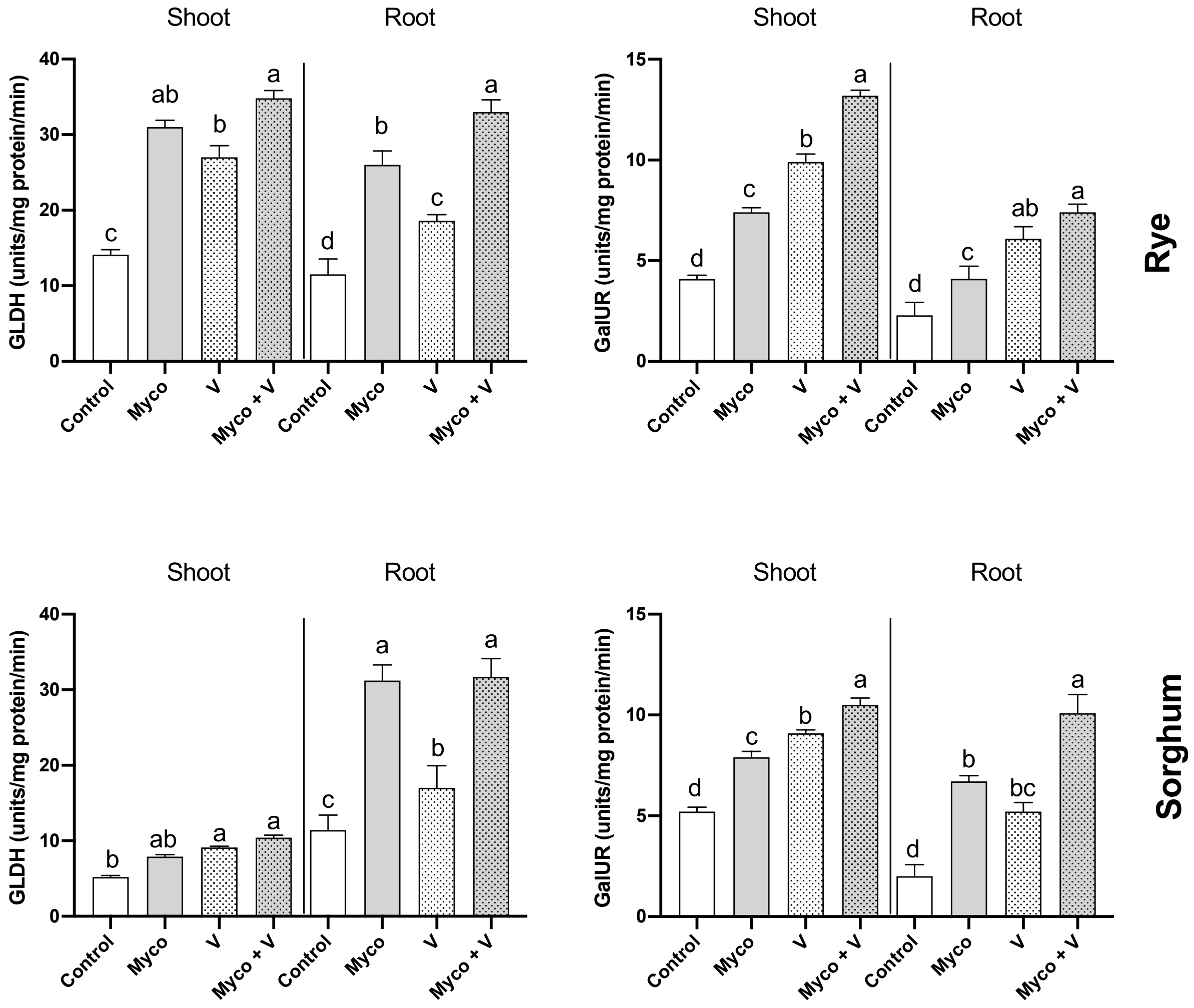

Supplementary Figure S2: Changes in the two key enzymes, GLDH and GalUR, involved in ascorbate metabolism in shoots and roots of 6-weeks old rye and sorghum grown in soils with 0 or 350 mg/kg soil sodium vanadate and with or without soil enrichment with inoculum of the mycorrhiza *Rhizophagus irregularis*. Data are mean values ± SE (n=3). Data were statistically analyzed by one-way ANOVA followed by Tukey posthoc test for comparing the means. Different letters indicate statistically significant difference between means of the same plant species at significance level at least ($P \leq 0.05$).
